# Standardized Input Function for ^18^F-FDG PET Studies in Mice: A Cautionary Study

**DOI:** 10.1371/journal.pone.0168667

**Published:** 2017-01-26

**Authors:** Marie Meyer, Lucie Le-Bras, Philippe Fernandez, Paolo Zanotti-Fregonara

**Affiliations:** 1 Department of Nuclear Medicine, Pellegrin Hospital, Bordeaux, France; 2 Aquitaine Institut for Cognitive and Integrative Neuroscience (UMR-5287), University of Bordeaux, Bordeaux, France; 3 Houston Methodist Research Institute, Houston, Texas, United States of America; University of Chicago, UNITED STATES

## Abstract

**Aim of the Study:**

The aim of this study was to assess the accuracy of a standardized arterial input function (SAIF) for positron emission tomography ^18^F-FDG studies in mice. In particular, we tested whether the same SAIF could be applied to populations of mice whose fasting conditions differed.

**Methods:**

The SAIF was first created from a population of fasting mice (n = 11) and validated within this group using a correlation analysis and a leave-one-out procedure. Then, the SAIF was prospectively applied to a population of non-fasting mice (n = 16). The SAIFs were scaled using a single individual blood sample taken 25 min after injection. The metabolic rates of glucose (CMRglc) calculated with the SAIFs were compared with the reference values obtained by full arterial sampling (AIF).

**Results:**

In both populations of mice, CMRglc values showed a very small bias but an important variability. The SAIF/AIF CMRglc ratio in the fasting mice was 0.97 ± 0.22 (after excluding a major outlier). The SAIF/AIF CMRglc ratio in the non-fasting mice was 1.04 ± 0.22. This variability was due to the presence of cases in which the SAIF poorly estimated the shape of the input function based on full arterial sampling.

**Conclusion:**

Although SAIF allows the estimation of the ^18^F-FDG mice input function with negligible bias and independently from the fasting state, errors in individual mice (as high as 30–50%) cause an important variability. Alternative techniques, such as image-derived input function, might be a better option for mice PET studies.

## Introduction

Positron Emission Tomography (PET) imaging in rodents is a fundamental step for evaluating new radioligands and for studying the effects of therapeutic agents.

Absolute quantification with PET often requires the catheterization of an artery to measure the plasma concentration of the tracer over time, i.e. the arterial input function (AIF). This procedure is particularly challenging in rats [1] and it is basically always avoided in mice, due to the small caliber of their arteries and because only a small volume of blood can be removed without altering physiological functions.

For ^18^F-FDG, various methods have been proposed to minimize blood sampling in small animals. The most common approach is to measure the input function directly from the PET images (image-derived input function, IDIF) using the time-activity curve of a blood pool, such as the heart [2–4]. This technique is however sensitive to partial volume effects and noise in the images, it requires a fast PET images acquisition protocol in order to maximize the chances to capture the peak of the arterial input function and, unless a data-driven algorithm is used, it is operator-dependent [5]. Recently, Lanz et al. validated an IDIF for mice using the activity of the inferior vena cava, measured by a new dedicated microPET device [6]. As demonstrated by several studies in humans, techniques validated using the physical parameters and acquisition characteristics of a particular scanner may not be easily transposable to different machines [7, 8].

A second approach is to insert a β+ probe into the femoral artery [9] or into an arteriovenous shunt [10] of the animal. This approach is rarely used, essentially because of the technical difficulty of the procedure. In addition, for those tracers that produce radiometabolites, blood samples cannot be analyzed to separate the plasma and to measure the parent concentration.

A third approach is using a standardized arterial input function (SAIF). This method is based on the assumption that the shape of plasma parent curves is constant among individual animals, and only the amplitude is different. The SAIF is thus scaled at the correct amplitude with one or two blood samples.

Compared to IDIF, SAIF does not suffer from artifacts related to the partial volume effect or noise in the images and does not require any image processing. This method was first proposed and validated in humans using ^18^F-FDG [11], and then expanded to several neuroreceptor tracers [12–15]. Of course, SAIF would work only if the injected bolus has a reproducible shape among subjects. This condition is verified if the injection is done under standard conditions and if different individuals have comparable metabolic status. Indeed, a SAIF obtained in a given population might not yield good results when applied to a population with a different metabolic condition or a disease [16].

A SAIF for ^18^F-FDG has also been successfully validated in rats [17]. Although at least one blood sample still needs to be acquired to scale the standard curve, the use of SAIF for rodent imaging would enable studies that would not be possible otherwise, because of the complexity of serial blood drawing in rodents and the limited amount of blood that can be withdrawn from the animal.

Mice, however, are smaller than rats. A bolus injection in smaller vessels is often difficult, and this might impact the reproducibility of the bolus. In addition, since ^18^F-FDG is an analogue of glucose, the kinetics of ^18^F-FDG may be influenced by how mice are fed before examination.

The aim of this study was to evaluate the bias and variability of a SAIF in ^18^F-FDG PET exams in mice, and its sensitivity to the fasting state of the animal.

## Methods

### Animal data

Arterial input functions and brain time-activity curves were obtained from the database Mouse Quantification Program of the Department of Molecular and Medical Pharmacology at the University of California, Los Angeles (UCLA) (http://dragon.nuc.ucla.edu/mqp/). This database includes male mice of the C57 Black 6 strain, aged 4 months on average (range: 2–12 months). Some of these mice were fed *ad libitum*, and some were fasted for 16 to 18 hours on average before imaging. The fed mice were not deprived of food until about 25 to 35 minutes before imaging (Dr. Wong, personal communication).

Mice were anesthetized with 2% isoflurane and manually injected with a ^18^F-FDG bolus through a catheter placed in a lateral tail vein. The injection was made over a constant time and usually completed in 4–8 seconds (Dr. Wong, personal communication). Blood samples were then manually drawn from the femoral artery for the duration of the acquisition of PET images. Dynamic images were obtained with a microPET Focus 220 (Siemens Preclinical Solutions, Knoxville, TN) [3, 18].

### Creation of the standard input function

All ^18^F-FDG input functions available in the database (thirty-seven) were downloaded. Then, we selected the input functions of better quality by eliminating those that were composed of less than 10 samples, those that lasted less than 60 minutes and those whose peak was not completely identified, either because the first measure already corresponded to the maximum activity (and therefore there was the possibility that the true peak had occurred before the first sample) or because no peak was clearly visible. In total, our study was based on 27 mice (11 fasting and 16 non-fasting).

The SAIF was first created using the population of fasting mice and validated within this group using a leave-one-out procedure. The fasting group was chosen to create the SAIF because the glucose level and metabolic status of these mice might be more reproducible across individuals. Then the SAIF was applied prospectively to the group of non-fasting mice to study the robustness of the technique vis-à-vis of a different feeding condition.

The input functions were first shifted to match the time of the peak, so that the final peak would represent the average of the individual peaks, and then averaged. A SAIF was also calculated using the median, rather than the average, of the individual values. The SAIF obtained from the median values was however very similar to that obtained from the average (difference of the area under the curve <1%). Therefore, all analyses were done with the average SAIF.

To overcome the error associated with slightly different sampling times, we fitted all input functions with linear interpolation until the peak and a tri-exponential function after the peak, and interpolated the samples on a grid of standard time points.

The SAIF was first validated in fasting mice, using a leave-one-out procedure [17]: to compute the SAIF of a mouse, we averaged the AIF of all mice except that of the mouse under study. This allowed us to avoid the bias of using a SAIF that includes the AIF to be estimated.

The SAIF were scaled with one or two blood samples. The optimal time point for the sample(s) was determined using a correlation analysis between the measured activity (or the average of the measured activities in the two samples, covering all possible combinations) and area under the curve calculated by the trapezoidal rule [11]. The time(s) that gave the highest Pearson’s correlation coefficient were chosen to calculate the scaling factor. These blood samples are those that are better correlated with the area under the curve of the input function.

### Image analysis

**Fig 1:**
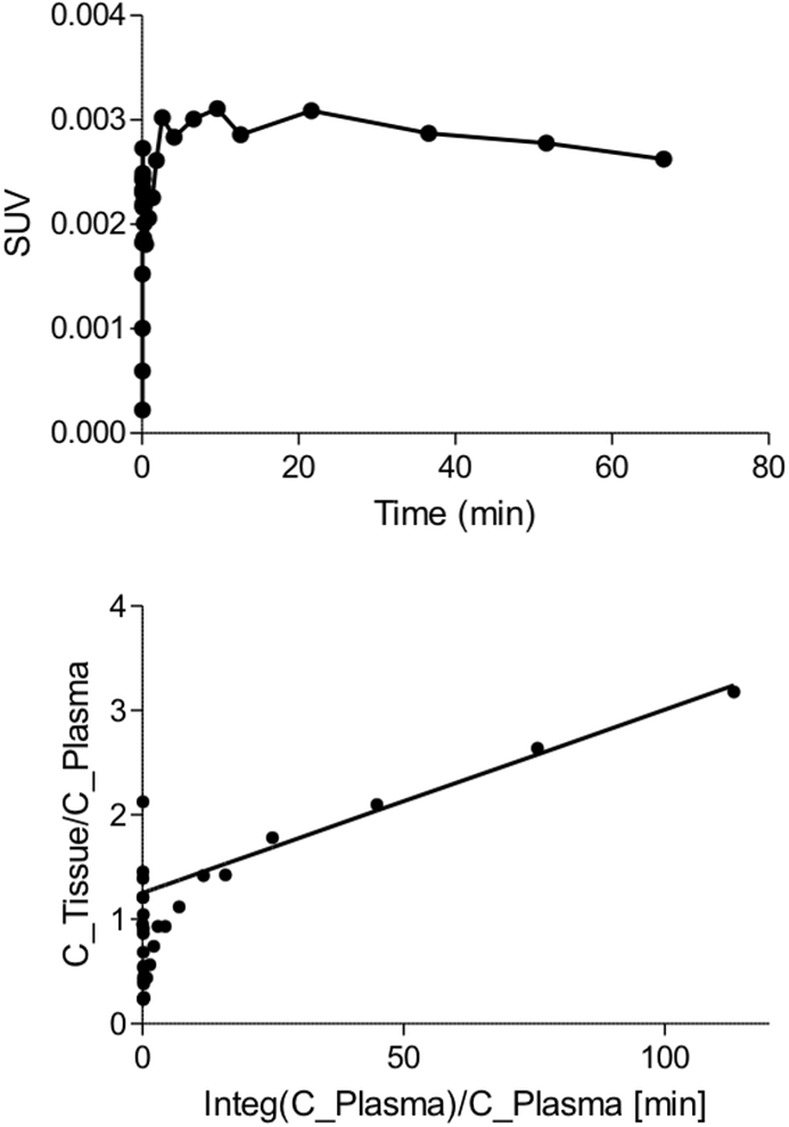
(A) Average brain time-activity curve used to quantify CMRglc. (B) Example of Patlak linearization using a scaled SAIF.

Brain time-activity curves were obtained by manually drawing a region of interest over the whole brain of the mice in the PET images. We selected the fifteen least noisy curves and averaged them to obtain a relatively smooth single brain curve, which was used for all mice (Fig 1A). This was done to make sure that any error found during kinetic modeling was due only to errors of input function estimation and not to poor fitting of the input function on a noisy brain curve. Please note that the use of a common brain curve has no impact on the assessment of bias and variability, since SAIF results are expressed as percentage difference against the reference AIF results. For both SAIF and AIF, we calculated the cerebral metabolic rate of glucose (CMRglc) with the graphical analysis of Patlak [19] (Fig 1B). Kinetic modeling and image processing were done with Pmod (Pmod Technologies, Switzerland).

## Results

The highest correlation coefficients were obtained either for a sample taken 25 minutes after injection (r = 0.997) or a combination of two samples at 20 and 37.5 minutes (r = 0.998) (Table 1). Since this small difference between the two coefficients did not justify the use of an additional blood sample, all SAIFs have been scaled using a single sample taken at 25 minutes.

**Table 1:** Coefficients of correlation between the radioactivity concentration of plasma samples and the area under the curve of the full arterial input functions.

Sample time (min)	0.23	0.3	0.7	1.5	2	8.3	15	20	25	30	37.5	45	55	65
0.23	0.767	0.745	0.827	0.816	0.814	0.804	0.799	0.796	0.794	0.792	0.789	0.787	0.784	0.781
0.3		0.390	0.528	0.530	0.529	0.496	0.487	0.484	0.480	0.477	0.471	0.465	0.458	0.451
0.7			0.830	0.827	0.528	0.830	0.884	0.889	0.881	0.897	0.907	0.913	0.917	0.921
1.5				0.933	0.530	0.884	0.933	0.939	0.939	0.955	0.964	0.970	0.975	0.979
2					0.943	0.889	0.939	0.943	0.942	0.960	0.969	0.976	0.980	0.985
8.3						0.929	0.939	0.942	0.929	0.953	0.966	0.975	0.981	0.987
15							0.973	0.960	0.953	0.973	0.983	0.990	0.994	0.997
20								0.991	0.995	0.997	0.998	0.996	0.992	0.987
25									**0.997**	0.990	0.995	0.997	0.997	0.995
30										0.995	0.997	0.997	0.995	0.990
37.5											0.980	0.995	0.990	0.980
45												0.952	0.982	0.968
55													0.901	0.990
65														0.841

The correlation coefficients for a single sample are shown in the diagonal of the table. The coefficients for two samples, covering all possible combinations, are above the diagonal. The highest values were obtained for a combination of two samples at 20 and 37.5 minutes (0.998) and for a single sample at 25 minutes (0.997). The latter was used to scale the SAIF curves.

**Fig 2:**
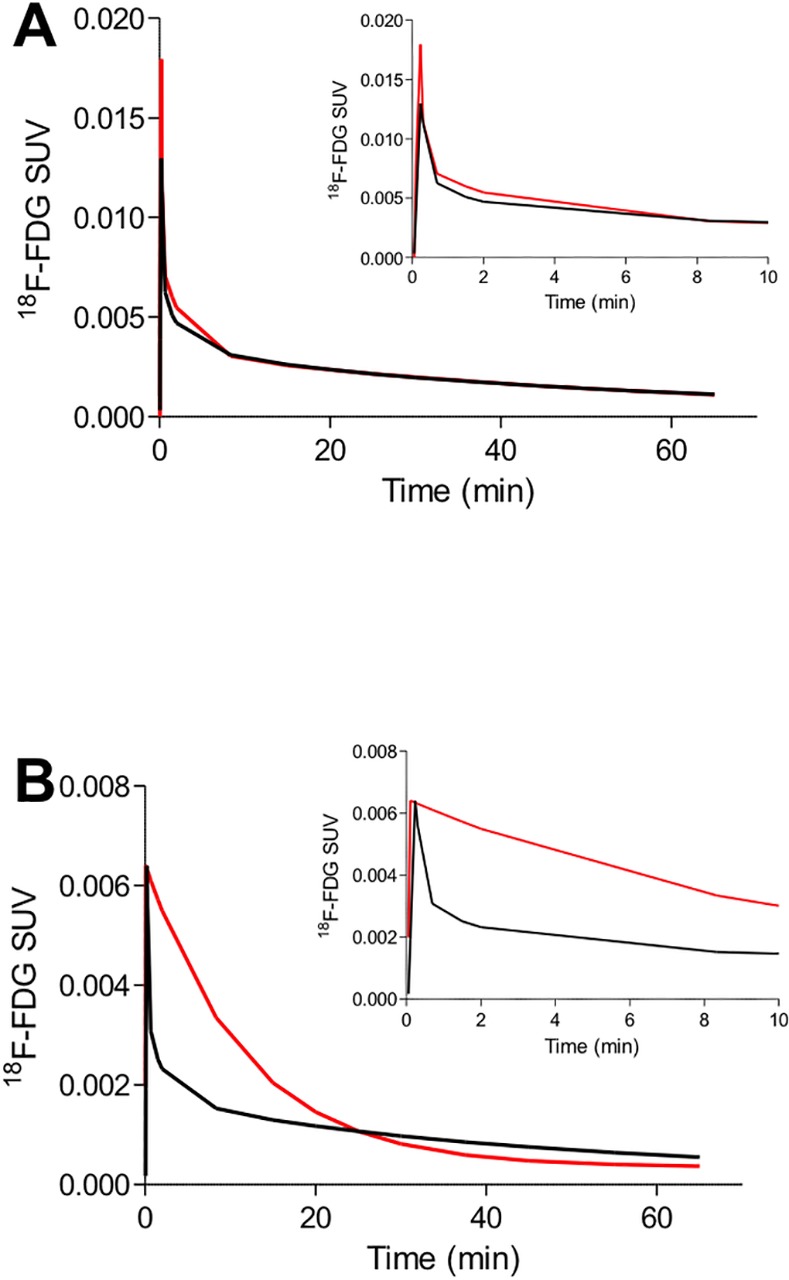
AIF (red) and SAIF (black) in two non-fasting mice. (A) Example of SAIF curve which is very similar to the blood reference curve (m17464). This SAIF is representative of the majority of the curves obtained in this study. There is however a significant number of AIF curves whose shape is incorrectly estimated by SAIF. (B) Example of AIF curve whose shape is incorrectly estimated by SAIF (m10911). One of the possible reasons is the difficulty of obtaining a reproducible bolus in the small tail vessels of mice. The insets magnify the first 10 minutes of the input functions.

Visually, the peaks were often over- or underestimated but in general the SAIFs reproduced well the second part of the input function (Fig 2A). In several mice, however, the shape of the SAIF was significantly different than that of the AIF, especially around the peak (Fig 2B).

The average bias of CMRglc quantification was negligible in both populations of mice but, due to the presence of several outliers in both populations, the variability was significant (8/11 fasting mice and 11/16 non-fasting mice had a CMRglc quantification error >10%). Five mice in total had an estimation error of at least 30% (Table 2).

**Table 2:** Ratio between the CMRglc values obtained with SAIF and the reference values obtained with AIF. Both populations of mice (fasting and non-fasting) show numerous estimation errors.

Fasting		Non-fasting	
Mouse	SAIF/AIF ratio	Mouse	SAIF/AIF ratio
m17156	0.78	m10911	0.95
m17884	3.07	m10948	1.02
m17973	0.62	m11082	1.43
m18333	1.30	m11122	1.10
m18482	0.95	m11467	0.99
m18519	1.05	m17332	0.87
m19481	1.16	m17385	0.81
m19755	0.92	m17437	1.07
m19796	1.26	m17464	1.03
m20108	0.80	m17540	0.88
m20117	0.87	m17709	0.86
		m17822	0.78
		m18714	1.34
		m18747	1.49
		m18778	1.11
		m18825	0.81

The SAIF/AIF CMRglc ratio in the fasting mice was 0.97 ± 0.22, average percentage of error: 0.18 (after excluding a major outlier). The AIF/AIF CMRglc ratio in the non-fasting mice was 1.04 ± 0.22, average percentage of error: 0.16. One mouse was eventually not included in the final results. For this mouse, the CMRglc value estimated with SAIF was about three times higher than those obtained with AIF. This high error was likely due not to a bad estimation of the area under the input curve (the SAIF/AIF area-under-the-curve ratio showed a difference of <5%), but to a poor identifiability of the reference Patlak values: the CMRglc standard error was 22.5% with AIF and 5.3% with SAIF. The standard error is expressed in percent of the parameter value and it is calculated as the square root of the diagonal elements in the covariance matrix.

## Discussion

This study shows that using a SAIF scaled with a single blood sample to quantify ^18^F-FDG dynamic PET images of mice produces unbiased CMRglc estimations compared to the reference results obtained with AIF. These results hold also when the same SAIF is used in populations of mice with different fasting conditions (the average bias was <5% for both fasting and non-fasting mice).

There was however an important variability in the estimation of individual CMRglc values in both populations, which was due to the presence of several outliers. Indeed, 19 out of 27 mice had a CMRglc error greater than 10%. These quantification errors were the consequence of a poor estimation of the shape of the input function, especially during the rapidly changing early portion of the curve.

It should be noted that in this study we carefully optimized the methodological approach to obtain the best possible SAIF results. First, we chose the best time of blood sampling by performing a correlation analysis between the values of the blood samples and those of the areas under the curve[11]. Notably, this preliminary step could improve the poor SAIF results obtained in some studies where it was not performed [15]. Second, we used an average brain curve, in order to minimize the errors due to noisy fitting. Errors are likely to be amplified if noisier curves are used, like those derived from regions of interest smaller than the entire brain. Third, we performed kinetic modeling with a Patlak graphical plot, instead of compartmental analysis. For Patlak analysis, the correct estimation of the area-under-the-curve is more important than the estimation of the precise shape of the input function, and therefore it is more robust with respect to time shift, dispersion, and errors in the estimation of the peak [5, 20]. In particular, the use of compartmental modeling (or spectral analysis) is associated with a larger variability of results when alternative input function techniques are used [5, 21, 22]. Fourth, these mouse PET examinations have undergone a careful quality control before being included in the Mouse Quantitation Project database. Fifth, we further restricted the analyses to the input functions of higher quality (see methods section). Therefore, it is likely that these results could be even worse if SAIF is applied to a population of unselected consecutive mice.

To our knowledge, Meyer et al published the only paper that attempted to quantify PET images with SAIF in rodents, but using rats instead of mice [17]. The results obtained with SAIF had a comparable bias but a much lower variability than our own. The average difference between the CMRglc calculated using the measured AIF and the SAIF scaled with one sample was 1.31 ± 5.45%. We attribute this discrepancy to the difference of size between rats and mice. Indeed, even if there are no obvious extravasations and a tail catheter is used, the very small size of mice tail veins may make the intravenous injections technically more difficult and thus reduce the reproducibility of the bolus.

Creating a SAIF by using data from a mixed population of fasting and non-fasting animals would not improve the results. Indeed, the poor results obtained in this study are not due to a systematic bias in one of the two populations (which can be minimized by mixing the two populations) or by effects of non-steady state glucose metabolism, but to the presence of numerous mice in both populations whose input function differed significantly from the SAIF template.

Tantawy and Peterson used this same dataset to validate an IDIF by using the combined curves from the heart and the liver of the mice, eventually normalized with a blood sample [3]. Although IDIF has drawbacks linked to the poor resolution of PET scanners and the noise in the images [5], their results were much more accurate than ours, in particular with smaller percentage errors [3]. Similarly, also using this same database, Mu and colleagues showed that spectral clustering could automatically extract the time-activity curve of cardiac tissue components with high accuracy and reproducibility [23]. The likely reason is because IDIF can track boluses with different shapes better than SAIF can do, and therefore IDIF should be the preferred approach to extract the input function from mice PET studies.

Although this study focused only on ^18^F-FDG, SAIF can be also applied to tracers that require metabolite-correction. In that case, the SAIF would be derived by averaging the parent concentrations in plasma and the scaling samples would have to be analyzed by high-performance liquid chromatography to separate the parent concentration from that of its radiometabolites. This approach has already been successfully validated in humans [24, 25], but it is possible that mice might yield a higher variability due to poorly reproducible boluses. However, since this study only used ^18^F-FDG, its results must be considered specific only to ^18^F-FDG.

All mice in the present study were imaged under anesthesia, as it is common practice for the vast majority of rodent PET studies. In rare occasions, however, rodents may need to be imaged while awake [26, 27]. Although, to our knowledge, there is no experimental data on how anesthesia affects the input function of mice, it is conceivable that the use of a SAIF obtained under anesthesia might add variability if used to estimate the CMRglc values of awake animals. Toyama and colleagues showed that anesthesia impedes ^18^F-FDG uptake in mouse brain and affects ^18^F-FDG uptake in heart, and these effects differed depending on the type of anesthesia used [26]. However, the arterial input functions were not measured and therefore we do not know whether changes in the input function contributed to these effects. Similarly, Itoh et al. showed that the ^11^C-(*R*)-rolipram B_max_ and K_D_ of conscious rats were significantly greater than those of anesthetized rats [27]. Although serial arterial sampling was performed in this study, the authors did not specify whether the input functions differed between the two groups of rats. Interestingly, Hines and colleagues reported a decrease of the total volume of distribution of ^11^C-PBR28 in the brain of healthy volunteers under propofol, but also a 13% increase of radioligand concentration in plasma [28].

In summary, although SAIF seems a practical way to perform full quantitative PET imaging in mice, this technique might entail large individual errors. This limitation ought to be properly taken into account when considering the number of mice needed in PET studies, especially by calculating an appropriate sample size. Alternative techniques, such as image-derived input function, might be a better option for mice PET studies.

## Conclusion

SAIF is a convenient alternative to the serial sampling needed for PET rodent studies with ^18^F-FDG. However, although average quantification results are generally unbiased, boluses are poorly reproducible in mice. Alternative techniques, such as image-derived input function, might be a better option for mice PET studies.
